# Defining the pig microglial transcriptome reveals its core signature, regional heterogeneity, and similarity with human and rodent microglia

**DOI:** 10.1002/glia.24274

**Published:** 2022-09-19

**Authors:** Barbara B. Shih, Sarah M. Brown, Jack Barrington, Lucas Lefevre, Neil A. Mabbott, Josef Priller, Gerard Thompson, Alistair B. Lawrence, Barry W. McColl

**Affiliations:** ^1^ The Roslin Institute Royal (Dick) School of Veterinary Studies, University of Edinburgh Midlothian UK; ^2^ UK Dementia Research Institute The University of Edinburgh, Edinburgh Medical School, The Chancellor's Building Edinburgh UK; ^3^ Centre for Clinical Brain Sciences University of Edinburgh Edinburgh UK; ^4^ Centre for Discovery Brain Sciences University of Edinburgh Edinburgh UK; ^5^ Department of Psychiatry and Psychotherapy Klinikum rechts der Isar, Technical University Munich Munich Germany; ^6^ DZNE Charité—Universitätsmedizin Berlin Berlin Germany; ^7^ Scotland's Rural College (SRUC) Edinburgh UK

**Keywords:** cross‐species, macrophage, microglia, pig, signature, transcriptome

## Abstract

Microglia play key roles in brain homeostasis as well as responses to neurodegeneration and neuroinflammatory processes caused by physical disease and psychosocial stress. The pig is a physiologically relevant model species for studying human neurological disorders, many of which are associated with microglial dysfunction. Furthermore, pigs are an important agricultural species, and there is a need to understand how microglial function affects their welfare. As a basis for improved understanding to enhance biomedical and agricultural research, we sought to characterize pig microglial identity at genome‐wide scale and conduct inter‐species comparisons. We isolated pig hippocampal tissue and microglia from frontal cortex, hippocampus, and cerebellum, as well as alveolar macrophages from the lungs and conducted RNA‐sequencing (RNAseq). By comparing the transcriptomic profiles between microglia, macrophages, and hippocampal tissue, we derived a set of 239 highly enriched genes defining the porcine core microglial signature. We found brain regional heterogeneity based on 150 genes showing significant (adjusted *p* < 0.01) regional variations and that cerebellar microglia were most distinct. We compared normalized gene expression for microglia from human, mice and pigs using microglia signature gene lists derived from each species and demonstrated that a core microglial marker gene signature is conserved across species, but that species‐specific expression subsets also exist. Our data provide a valuable resource defining the pig microglial transcriptome signature that validates and highlights pigs as a useful large animal species bridging between rodents and humans in which to study the role of microglia during homeostasis and disease.

## Main Points

- Defined a pig microglial transcriptome signature comprising 239 genes.
- Demonstrated regional variance in the pig microglial transcriptome across the brain.
- Revealed similarity of pig microglial gene expression to that of human and mouse microglia.

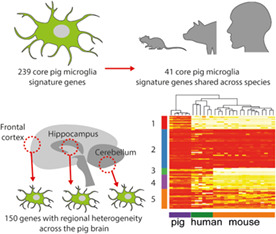

## 1 INTRODUCTION

Microglia are resident mononuclear phagocytes of the central nervous system (CNS) parenchyma that are increasingly recognized to play an important role in the development, homeostasis and diseases of the CNS (Li & Barres, 2018).

Microglia are derived from erythro‐myeloid progenitors during early embryonic development (Reemst et al., 2016) and are not replenished by blood monocytes under normal physiological conditions (Gomez Perdiguero et al., 2015). Microglia sense changes in their environment through a large repertoire of receptors, and mediate responses that promote neuronal and synaptic health, and assist in tissue protection and repair to microbial and sterile injury stressors (Hickman et al., 2018). However, in specific contexts some phenotypes of microglia are thought to contribute to disease processes including in neurodegenerative disease. Indeed, reactive microglia and inflammatory cytokines are commonly observed around lesions in several neurodegenerative disorders, including Alzheimer's disease (Frautschy et al., 1998), Parkinson's disease (McGeer et al., 1988), and multiple sclerosis (Kuhlmann et al., 2017). Age‐dependent changes in microglia activation and regulation have been reported in rodents (Ogura et al., 1994; Perry et al., 1993), nonhuman primates (Sheffield & Berman, 1998) and humans (Streit & Sparks, 1997), and have been associated with deficits in psychomotor coordination (Richwine et al., 2005) and cognitive function (Jang et al., 2010; Rosczyk et al., 2008) in mouse models of aging. Microglial reactivity is also associated with a wide range of psychosocial stressors (Calcia et al., 2016; Stein et al., 2017), and behavioral susceptibility to social stress is driven by microglial induced increases in reactive oxygen species (Lehmann et al., 2019).

Microglia have a distinct transcriptome from tissue‐resident macrophages in other organs and from the other cell types in the CNS (Butovsky et al., 2014). A number of transcriptomic studies have characterized the gene expression signature for microglia in non‐neuropathologic individuals in humans and mice (Bennett et al., 2016; Butovsky et al., 2014; Darmanis et al., 2015; Galatro et al., 2017; Hawrylycz et al., 2012; Sankowski et al., 2019). These microglial gene signatures have been instrumental to understanding the core molecular identity of microglia, their diversity, and as a basis to characterize the spatiotemporal transcriptional changes of microglia in response to aging and disease conditions (Hammond et al., 2019; Patir et al., 2019). Microglial studies have generally been conducted in rodent models or human post‐mortem brain tissue. While many cross‐species similarities are evident, differences have also been described (Galatro et al., 2017; Chen & Colonna 2021), and may have implications when extrapolating findings across different animal species. Significant differences may also be evident when comparing species such as mice and humans due to substantial disparities in body weight, brain mass and lifespan, which can be partly mitigated in some large animal species.

Pigs are a physiologically relevant animal model for studying human neurological disorders. In contrast to rodents, pigs have large human‐like gyrencephalic brains and human‐like gray:white matter ratio. These anatomic features are ideally suited for neuroimaging, cell transplantation and gene therapy studies (Lind et al., 2007; Sauleau et al., 2009; Simchick et al., 2019; Sjostedt et al., 2020). Recent data from Sjostedt et al. (2020) suggested that the global gene expression profiles for some brain regions (such as the cerebellum and hypothalamus) in pigs were more similar to those of humans, than those of mice to humans (Sjostedt et al., 2020). However, the transcriptome‐wide signature that specifically defines microglia in the pig brain is poorly defined. As microglia are the principal cellular mediators of innate immunity in the CNS, it is relevant to note previous studies indicating the greater similarity of pigs (than rodents) to humans in certain aspects of innate immune physiology, notably macrophage activation signaling (Fairbairn et al., 2011; Kapetanovic et al., 2012). Transcriptional analyses of pig mononuclear phagocytes have suggested their responses are more similar to human cells than those from mice (Robert et al., 2015) e.g. pigs and other large mammals differ from mice in their ability to induce the expression of genes responsible for arginine metabolism and nitric oxide production (Bush et al., 2020). Furthermore, microglia are thought to be instrumental in mediating responses to non‐disease challenges such as social stress (Mondelli et al., 2017; Salter & Stevens, 2017). With the increasing recognition of the pig as an intermediate species for translational biomedical research (Lunney et al., 2021), it is important to establish a normative microglial profile in pigs and instructive to relate this to signatures in other species. In addition, pigs are one of the most economically important and intensively farmed livestock species, and a better molecular definition of pig microglia may aid understanding of conditions that can promote their health, welfare and productivity. Our previous work implicated microglia in the effects of environmental enrichment on neural health including altered microglial gene expression in pigs provided with enrichment (Brown et al., 2018). The aims of this study were to define the transcriptome identity of pig microglia, and conduct comparative analysis across brain regions, and with mouse and human microglia signatures.

## 2 METHODS

### 2.1 Ethical review

All work was carried out in accordance with the UK Animals (Scientific Procedures) Act 1986 under EU Directive 2010/63/EU following ethical approval by SRUC (Scotland's Rural College) Animal Experiments Committee. All routine animal management procedures were adhered to by trained staff and health issues treated as required. All piglets remaining at the end of the study were returned to commercial stock.

### 2.2 Animals and general experimental procedures

Sixteen commercial cross‐bred female breeding pigs (sows; Large White × Landrace) were artificially inseminated using commercially available pooled semen (Danish Duroc). Piglets were born into either standard commercial housing or pens, allowing greater behavioral freedom (Baxter et al., 2011). Sows were balanced for parity across both conditions. No tooth resection was performed and males were not castrated. In line with EU Council Directive 2008/120/EC tail docking was not performed. In accordance with the Defra Code of Recommendations for the Welfare of Livestock, temperature within the room was automatically controlled at 22°C and artificial lighting was maintained between the hours of 0800 to 1600, with low level night lighting at other times. At around 21 days of age small amounts of weaning diet (ForFarmers Ultima 2) was introduced to the piglets. At between 24 and 26 days of age, one male piglet (7–8 kg) per litter was selected for tissue collection. The piglets used in this study were part of a wider study involving in vivo neuroimaging by MRI under sedation. Piglets were sedated with a combination of ketamine (5 mg/kg), midazolam (0.25 mg/kg) and medetomidine (5 μg/kg) injected intra‐muscularly (quadriceps). After 3–5 min when profound sedation was present, anesthesia was induced with 2%–3% isoflurane delivered by a Hall pattern mask until adequate jaw relaxation allowed laryngoscopy and the topical application of 0.8–1.0 ml 2% lidocaine solution. Endotracheal intubation was conducted using a 5 mm OD endotracheal tube 90 s later. Anesthesia was maintained with isoflurane in O_2_ delivered using a Bain breathing system. The lungs were inflated mechanically to produce normocapnia. Core temperature was maintained using hot air blowers. The animal was euthanized humanely under anesthesia using pentobarbital IV (40 mg/kg) iv.

### 2.3 Tissue collection

Piglet brains were removed and then cut into two hemispheres. All dissections were performed by a single experienced researcher using http://www.anatomie-amsterdam.nl/sub_sites/pig_brain_atlas for reference, utilizing both parasagittal and rostrocaudal views. One hemisphere was dissected into broad anatomical regions for microglial isolation and tissue RNA extraction. Samples for microglial isolation were minced in 1× HBSS (w/o Ca^2+^ and Mg^2+^, 12 mM HEPES) and placed on ice for immediate cell isolation. Adjacent samples for tissue level RNA extraction were placed in RNAlater at room temperature for 30 min then snap frozen on dry ice and stored at −20°C until required. The average time from confirmation of death to tissue being stored in RNAlater was approximately 8.5 min. The opposite hemisphere was placed directly into 4% PFA for later histological analysis. After 10 days, the solution was changed for Tris Azide and samples maintained at 4°C. Alveolar macrophages were collected in saline solution by post‐mortem bronchoalveolar lavage (<5 min from confirmation of death) and placed on ice for immediate cell isolation (further 1–2 min).

### 2.4 Preparation of brain cell suspensions

We adapted methods previously described for rodent microglial isolation (Grabert & McColl, 2018). Samples of frontal cortex, hippocampus and cerebellum in 1XHBSS buffer were transferred individually to a glass Dounce homogenizer on ice and cells dissociated by 40 passes of the pestle. Cells were pelleted by centrifugation and resuspended in 35% isotonic Percoll over ice. The sample was overlaid with 1XHBSS without disrupting the Percoll gradient and spun at 4°C for 45 min. The separated myelin layer and supernatant were removed and the cell pellet resuspended in 10 ml HBSS. Cell suspensions were filtered through a 70 μm filter and pellets resuspended in FACS (fluorescence‐activated cell sorting) buffer (1× PBS, 25 mM HEPES, 0.1% BSA).

### 2.5 Isolation of microglia and alveolar macrophages

Brain and alveolar lavage cell suspensions, were incubated with 1% Human IgG1 Fc block (R&D systems) for 30 min. Samples were centrifuged, supernatant removed and cells resuspended in a combination of mouse anti‐human CD11b:Pacific Blue (Biolegend) and mouse anti‐pig CD45:AF647 antibodies (BioRad) for microglial isolation, or mouse anti‐pig CD163:FITC (Biorad) and F4/80:AF647 (mouse anti‐pig ADGRE1; ROS‐4E12‐3E6) (Waddell et al., 2018) antibodies for alveolar macrophage isolation for 30 min incubation at room temperature. Samples were centrifuged, supernatant removed and cells resuspended in FACS buffer. Samples were sorted on a FACSAria III cell sorter. Microglia in brain cell suspensions were identified as CD11b^hi^CD45^lo^ and macrophages identified from the alveolar lavage suspensions as F4/80^+^CD163^lo^. Sorted cells were collected directly into TRIzol reagent (Invitrogen) for immediate RNA isolation.

### 2.6 Microglial and macrophage RNA extraction

Cell suspensions were transferred into 2 ml lysing matrix D tubes (MP Biomedicals) and homogenized on a FastPrep 24 at 6.5 m/s for 50 s. Homogenate was transferred to a 1.5 ml microtube and incubated at room temperature for 5 min to allow complete dissociation of nucleoprotein complexes. An equal volume of chloroform was added to the homogenate, samples placed on a shaker at room temperature for 5 min, and then centrifuged at 12,000 rmp, 4°C for 15 min. The 100 μl of the resulting aqueous phase was transferred to a 96 well plate and 60 μl isopropanol added. The plate was placed on an orbital shaker at medium speed for 1 min. Twenty microliters of MagMaxTM beads were added to each well and plate returned to orbital shaker for 3 min. The 96‐well‐plate was placed onto a magnetic separation rack and supernatant removed without disturbing the magnetic beads. This was repeated until all the aqueous phase had been used. The remaining extraction was performed as per the MagMaxTM −96 total RNA isolation manufacturers protocol, including a TURBOTM DNase clean up step, with a final total RNA elution volume of 40 μl.

### 2.7 Hippocampus whole tissue RNA extraction

One hundred milligrams of RNAlater‐stabilized tissue was homogenized in 1 ml QIAzol reagent using a Qiagen TissueRuptor II on a medium speed setting for 40 s, or until the lysate was uniformly homogeneous. Total RNA extraction was performed as per the Qiagen RNeasy Lipid tissue mini kit product guidelines, to a final elution volume of 40 μl.

### 2.8 RNA quantification and quality control

Quantification and quality control of RNA samples were performed on an Agilent 4200 Tapestation. Tissue samples returned Total RNA with concentrations of 80–150 ng/μl. Isolated cell suspensions returned total RNA with concentrations of 110–340 pg/μl. A RIN cut‐off was set at 6.0.

### 2.9 RNA sequencing and analysis

Samples were prepared for sequencing using the Takara SMARTer stranded total RNA‐Seq vs2 library prep protocol. Sequencing was performed as paired‐end reads with a read length of 50 bp. A total of 31 samples were analyzed from 16 pigs (multiple brain regions were sampled for 12 pigs) comprising alveolar macrophages (*n* = 3), microglia from cerebellum, frontal cortex, and hippocampus (*n* = 8 per region), and hippocampal whole tissue (*n* = 4). Raw sequence files (FASTQ format) were filtered and trimmed with BBtools (version 38.67) (Bushnell), followed by alignment to the Sus scrofa genome (Sscrofa11.1; Ensembl Release 99) using Hisat2 (Kim et al., 2015). Gene‐level counts were generated from the resulting BAM files using StringTie (Pertea et al., 2015), and normalized gene expression (FPKM) data were subsequently made with Ballgown (Frazee et al., 2015). Differential gene expression analysis was performed using Limma (Ritchie et al., 2015) on genes with >10 CPM (counts per million) in all samples of at least 1 subgroup. TMM normalization was used in EdgeR (Robinson et al., 2010). Differential gene expression was carried out using voom from Limma, using an adjusted *p*‐value cut‐off of 0.01. The fold change (FC) threshold for comparing hippocampal microglia against hippocampal brain tissue and microglia versus macrophages were set to 4 and 3, respectively, and no FC threshold was set for characterizing regional variations. The RNA‐seq data is available via Gene Expression Omnibus (GEO https://www.ncbi.nlm.nih.gov/geo/, accession number: GSE172284).

Gene expression in microglia isolated from frontal cortex, cerebella, and hippocampus was compared to those in macrophages, and from this, macrophage‐enriched genes were defined. The microglia‐enriched genes were derived using genes found to be more highly expressed in microglia relative to both macrophage and corresponding tissue (hippocampal microglia compared to hippocampus tissue). A microglial gene list was generated by refining the common DEG from the aforementioned gene expression comparisons through the use of mouse and human brain expression data from www.brainrnaseq.org (Bennett et al., 2016); genes were removed if they were expressed in nonmicroglia cell types (>2 FPKM) and were less than fourfold higher in microglia than the nonmicroglia cell types in the human or mice datasets. As some pig genes were not annotated with a gene name, human orthologues were used in addition to the pig gene names.

When examining regional variations, each pair‐wise comparison (cerebellum vs. frontal cortex, frontal cortex vs. hippocampus, hippocampus vs. cerebellum) was carried out. For each of the genes showing significant regional variation, the different brain regions were ranked according to their gene expression level and the region with the highest expression noted. Genes showing significant regional variation were filtered (to minimize inclusion of nonmicroglial‐expressed genes which may arise from minor contaminating cells) using mouse and human brain expression data from www.brainrnaseq.org (Bennett et al., 2016); genes with <2 FPKM in microglia and >2 FPKM in nonmicroglia cells in either mouse or human data were removed.

Gene–gene co‐expression network analysis was carried out using data for microglia isolated from cerebellum, frontal cortex, and hippocampus using Graphia Professional (version 3.0. Kajeka, Edinburgh, UK), with a Pearson correlation threshold of ≥0.84 on genes with ≥1FPKM in at least 1 sample, keeping components with a minimum size of 10, followed by Markov clustering (MCL) using an inflation value of 1.8. Gene Ontology (GO) enrichment analysis was carried out on both genes showing significant regional variations and annotations in the Pig Expression Atlas (Freeman et al., 2012); an adjusted *p*‐value <1 × 10^−5^ and a minimum of 10 genes were required for an enrichment to be considered significant. We carried out the co‐expression analysis for two reasons. First, it is more inclusive by including additional genes beyond those meeting statistical thresholds based only on pair‐wise filtering, thereby allowing larger gene sets to be used in the GO enrichment analysis. Second, if multiple processes were associated with a brain region, the genes involved in each process can be deconvolved, allowing for better defined input for GO enrichment analysis.

Sample‐sample network analysis examining the relationships of microglia isolated from the three brain regions was carried out in Graphia with log2 FPKM and a Pearson correlation coefficient threshold >0.94 on genes showing significant regional variation.

Functional enrichment was carried out using Metascape (Zhou et al., 2019), with the settings for min overlap, *p* value cut‐off and min enrichment set to 3, 0.01, and 1.5 respectively, using the Gene Ontology (GO) Biological Processes database.

### 2.10 Cross‐species comparison of microglial transcriptome signatures

Raw RNAseq data on isolated microglia from mice (*n* = 17) were obtained from Geirsdottir et al. (2019) (NCBI BioProject: PRJNA556201). Non‐pathologic human microglia RNAseq data were from 2 separate studies (*n* = 6; NCBI run IDs: SRR9909238, SRR9909237, SRR9909236, SRR6849268, SRR6849266, and SRR6849267) (Sankowski et al., 2019; van der Poel et al., 2019). These data were integrated with six of the pig microglia samples from this study (two from each brain region). Processing of data from raw FASTQ files to FPKM were the same as those used in processing the pig data for this study. Reference genomes and gene annotations for the respective species were downloaded from the Ensembl databases (Release 99). For each species, the orthologue genes matching to each human gene were noted using Ensembl BioMart (Ensembl Genes 99) (Kinsella et al., 2011). In cases where multiple orthologue genes mapped to the same human gene, the sum FPKM were used. We created a merged and normalized RNAseq dataset that contained annotated genes mapping across the three species using homologues that matched to the corresponding human gene in the BioMart database (GRCh38.p13).

Analysis was limited to genes with matching human homologues across all species. These genes were ranked according to their FPKM, followed by minmax normalization of their ranks to a value between 0 and 1, with 0 indicating the least expression and 1 the highest expression. Sample‐sample correlation was carried out using genes with >0.5 normalized expression value in at least one species, keeping edges with *r* > 0.35 and using a k‐nearest neighbor (knn) value of 6. Following this, the expression of microglia‐enriched genes was examined for four gene lists: pig microglia‐enriched genes (derived from this study), human microglia‐enriched genes (two gene lists from Galatro et al., 2017; Patir et al., 2019) and mouse microglia‐enriched genes (Butovsky et al., 2014). Two human microglia‐enriched gene lists were used to cover different methods deriving an enriched microglial gene signature; one from co‐expression (Patir et al., 2019) and the other from fold‐change enrichment (Galatro et al., 2017). For the gene list from Galatro et al. (2017), we included the additional filter on the microglia‐gene list provided in the manuscript, keeping only genes with adjusted *p* < 0.001 and FC >2 when comparing microglia to monocytes, and macrophages.

### 2.11 Single molecule mRNA in situ hybridization

Four micrometers of FFPE brain sections were labeled for *C3* transcript using the RNAscope 2.5 HD‐RED kit (ACD Biosciences) following manufacturer's recommended workflow with slight modifications to improve signal. For single labelling of *C3*, antigen retrieval and protease plus digestion was performed for 30 min. To co‐label IBA1, sections were fixed for 15 min at 4°C using 10% neutral buffered formalin following *C3* labelling. A second round of antigen retrieval was subsequently performed (15 min; white matter, 30 min; gray matter) using Tris‐EDTA pH 9. Anti‐IBA1 (1.2 μg ml^−1^, 019–19,741, Fujifilm Wako) was incubated overnight at 4°C and fluorescently labeled using tyramide (AF488; Thermofisher). DAPI (1 μg ml^−1^) was used to label nuclei. Due to white matter sensitivity to protease digestion, initial *C3* antigen retrieval and protease digest was restricted to 15 min each when co‐labelling with IBA1. All antigen retrieval steps were performed in pre‐heated solutions in a 97.5°C waterbath.

### 2.12 Microscopy and image analysis

Images were captured using a Zeiss Axioimager D2 microscope. Brightfield images were automatically white balanced. For *C3* transcript quantification, 5 nonoverlapping 40× ROIs were captured in outer regions of cerebellum and frontal cortex sections per animal and exported in OME‐TIFF formats (Zen Blue V3.4.91). Per cell transcript abundance was quantified using a customized CellProfiler (V4.2.1) pipeline available upon reasonable request. Briefly, color unmixing separated nuclei and transcripts, nuclei were segmented and cell boundaries expanded. Transcripts were detected and mapped to cells. Cells containing <20 transcripts were removed from downstream analysis as these were judged to be non‐*C3* expressing cells contaminated by microglial ramifications. Outer regions of each section were imaged due to low cellularity aiding nuclei segmentation and cell profiling. Regional expression (transcripts per cell) was compared between cerebellar and cortical microglia by paired *t*‐test. *p* < 0.05 was considered statistically significant.

## 3 RESULTS

### 3.1 Identification of comparative pig microglia and macrophage transcriptomes

**FIGURE 1:**
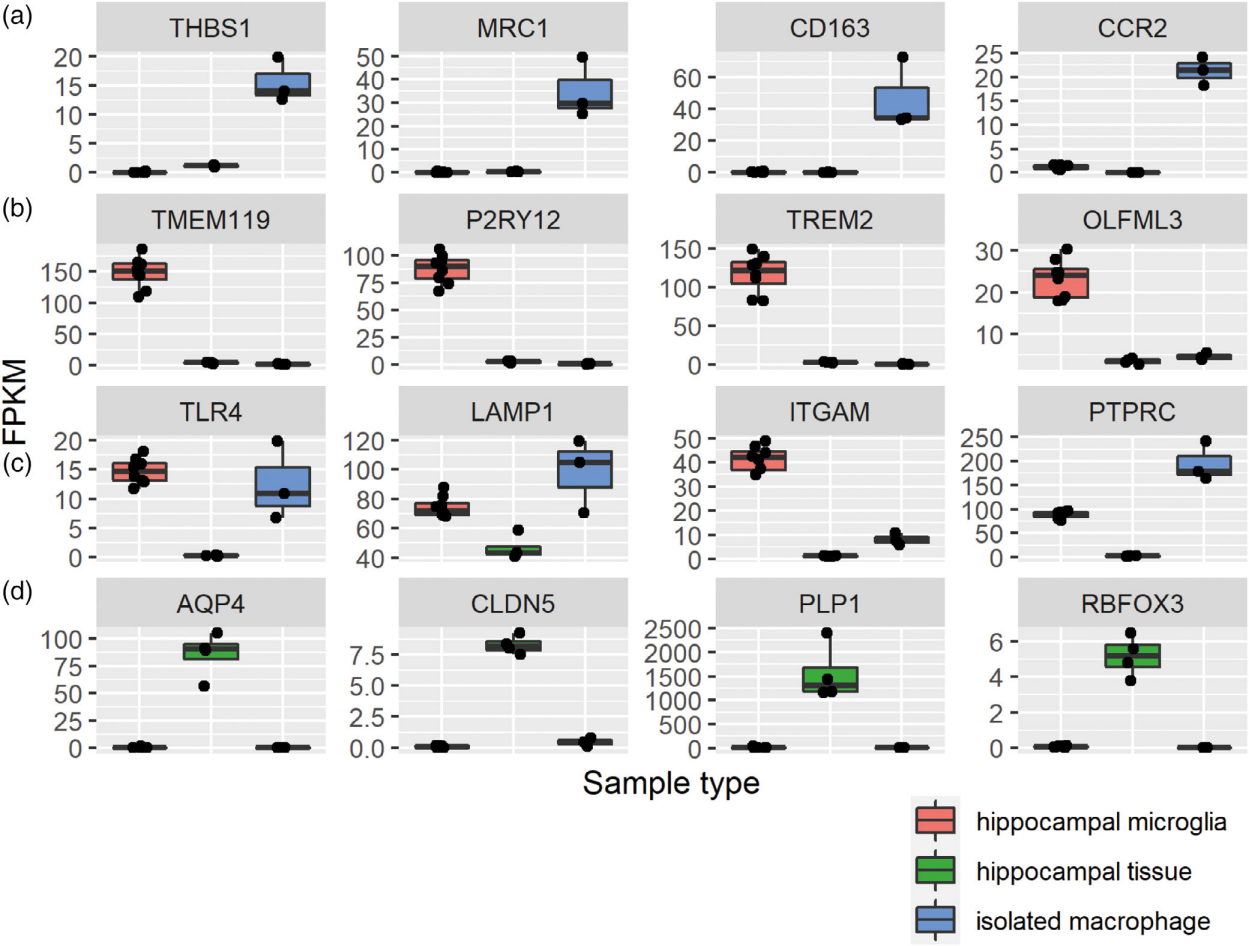
Specificity of cell isolation according to selective expression of canonical cell‐type genes in isolated cells and brain tissue. Expression of genes in our RNAseq dataset for established enriched genes from previous studies in rodents or humans for (a) non‐CNS monocytes/macrophages, (b) microglia, (c) myelomonocytic cells, (d) CNS cell types (AQP4, astrocytes; CLDN5, endothelial cells; PLP1, oligodendroglia, RBFOX3, neurons). The samples in the plots included alveolar macrophages (*n* = 3), hippocampal microglia (*n* = 8) and hippocampal whole tissue (*n* = 4).

We first defined the pig microglia‐enriched gene set by comparing the transcriptome of sorted microglia with isolated alveolar macrophages (as an exemplar systemic macrophage comparator) and whole hippocampal tissue both from the same animals. The pattern of expression for selected canonical genes enriched in microglia, other parenchymal CNS cell types, and CNS border/systemic macrophages provided initial validation of the specificity of the cell sorting procedure (CD11b^+^CD45^lo^ for microglia, F4/80^+^ for alveolar macrophages, Figure S1). Notably, microglial samples expressed high levels of genes established as enriched in microglia in other species and negligible levels of CNS border macrophage‐enriched genes (e.g., CD163, MRC1) that, in contrast, showed a reciprocal pattern in alveolar macrophages (Figure 1). Expression of archetypal neuronal, astrocytic, oligodendroglial, and vascular genes were also negligible in sorted microglial samples (Figure 1). These data confirm specificity of the cell sorting protocol for parenchymal microglia.

**FIGURE 2:**
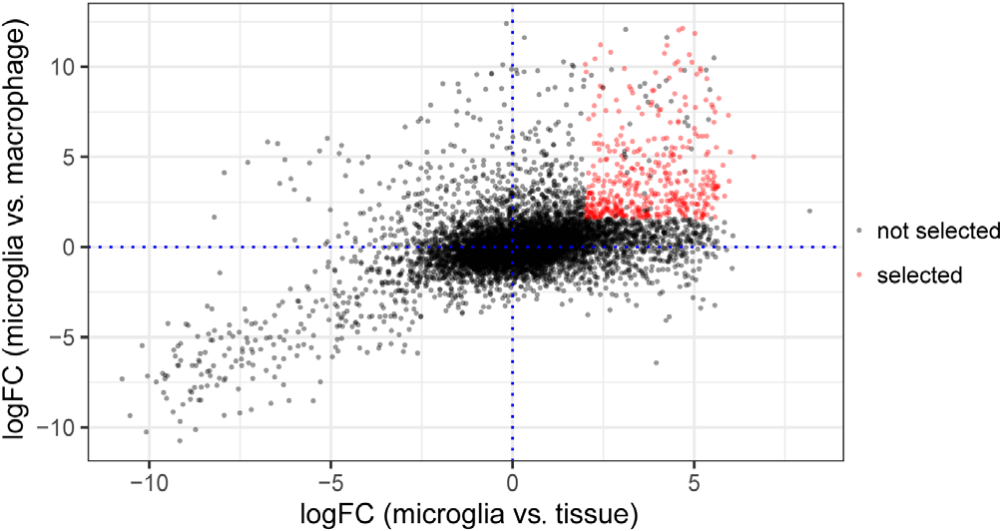
Selected genes from the two statistical comparisons. The scatter plot indicates the 434 genes meeting the adjusted *p*‐value (<0.01) and fold‐change criteria for both the hippocampal microglia (*n* = 8) versus hippocampal whole tissue (*n* = 4) (>4 FC) and the microglia (*n* = 24) versus alveolar macrophages (*n* = 3) (>3 FC) comparisons.

**FIGURE 3:**
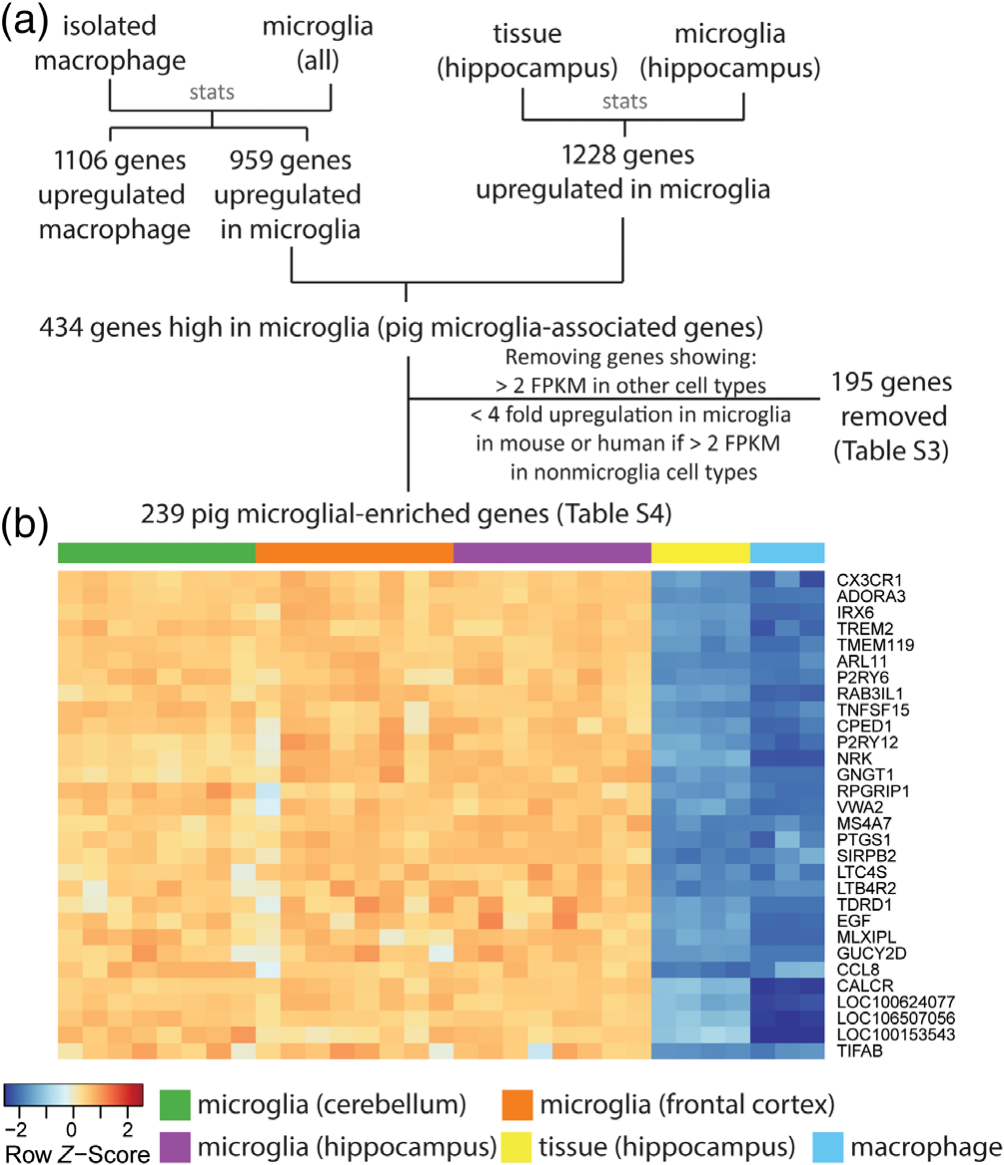
Derivation of the pig microglia gene expression profile. (a) The 959 upregulated differentially expressed genes (DEG) when comparing isolated microglia to macrophages have 434 genes in common with the 1228 upregulated DEGs when comparing isolated hippocampal microglia to hippocampal tissue. By using data from Brain RNAseq (https://www.brainrnaseq.org/), we excluded 195 genes (Table S3) showing <4‐fold upregulation in microglia and >2 FPKM expression (fragments per klobase of transcript per million) in human or mouse astrocytes, neurons, oligodendrocytes or endothelial cells from the 434 DEGs. This yielded a final 239 genes as the pig microglia‐enriched genes (Table S4). (b) The top 30 most enriched genes in microglia (with gene names) are shown in the heatmap (Table S5). *Z*‐score was used to represent the FPKM expression as standard deviations away from the mean for each gene. The samples in the plots included alveolar macrophages (*n* = 3), microglia (*n* = 8 for each region) and hippocampal whole tissue (*n* = 4).

Differentially expressed genes (DEG, adjusted *p* < 0.01) were used to define pig microglial signature genes, which are genes with high expression in microglia compared to alveolar macrophages (FC >3) and whole brain extracts from the hippocampus (FC >4). A total of 959 genes were more highly expressed (Table S1) in microglia versus macrophages, and 1228 genes were more highly expressed in isolated hippocampal microglia compared to hippocampal whole tissue (Table S2). Among these sets of DEGs, 434 were present in both comparisons (Figure 2). Although we confirmed high specificity of microglial sorts (see above) we reasoned that even a very minor contamination with other CNS cellular constituents could result in enrichment when compared to alveolar macrophages and potential aberrant inclusion in the microglial signature. We therefore refined the 434 gene list by cross‐checking (see Section 2) expression against all cell types in the Brain RNAseq dataset (http://www.brainrnaseq.org) (Bennett et al., 2016) (Table S3).This created a stringent set of 239 genes comprising the pig microglia‐enriched gene list (Tables S4 and S5, Figure 3). We observed that many genes previously shown to be microglial‐enriched genes across other species were also contained in the porcine list (e.g., *C3*, *CSF1R*, *CX3CR1*, *GPR34*, *OLFML3*, *P2RY12*, *TREM2*).

**FIGURE 4:**
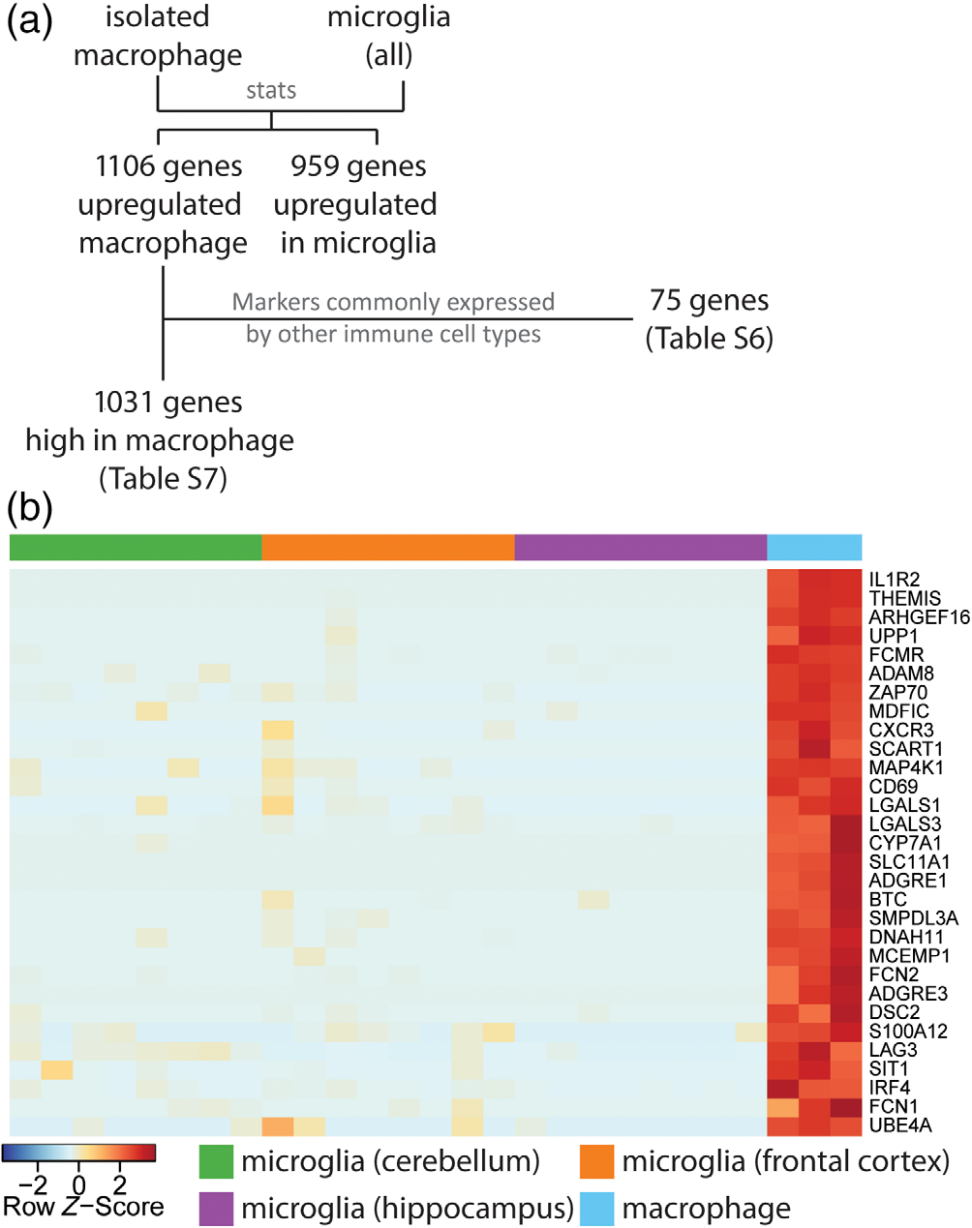
Genes enriched in isolated macrophages compared to microglia. (a) When comparing isolated macrophages to microglia, 1106 genes are upregulated in macrophages. Of these, 75 genes were markers commonly expressed by other immune cell types such as T‐cell, B‐cell, and plasma cells [58 genes in ImSig (Nirmal et al., 2018), e.g., *CD2*, *PVRIG*, *CD19*, *NLRC3*, *CD8A*; 17 genes are related to immunoglobulin], these are highlighted in Table S6. The remaining 1031 gene can be found in Table S7. (b) The heatmap illustrates the top 30 genes (with gene names) showing the highest enrichment in macrophages (Table S8). *Z*‐score was used to represent the FPKM expression as standard deviations away from the mean for each gene. The samples in the plots included alveolar macrophages (*n* = 3), microglia (*n* = 8 for each region).

We also assessed the genes that were enriched in macrophages compared to microglia to further understand the differential gene signatures. One thousand one hundred and six genes were significantly more highly expressed (adjusted *p* < 0.01, FC >3) in isolated alveolar macrophages compared to microglia. We further filtered these (see Methods) to minimize inclusion of genes that might arise from even negligible numbers of contaminating immune cells (e.g., T cells, B cells) in the alveolar samples by surveying expression across the major immune cell classes in the ImSig dataset (Nirmal et al., 2018) (Table S6). This produced a high‐stringency set of 1031 genes enriched in macrophages compared to microglia (Table S7, Figure 4). The top 30 macrophage‐enriched genes (highest FC and with gene annotation) were are all expressed at <1 FPKM in microglia samples (Figure 4, Table S8) showing they are both enriched for macrophages relative to microglia and indicating they could be a useful set of negative selection markers for studying pig microglia. In summary, we have created high‐stringency pig microglial and systemic (alveolar) macrophage gene signatures (Table S4), highlighting the distinct transcriptomic identities of these tissue macrophage populations in the pig (Table S7).

### 3.2 Regional variation of the pig microglial transcriptome

**FIGURE 5:**
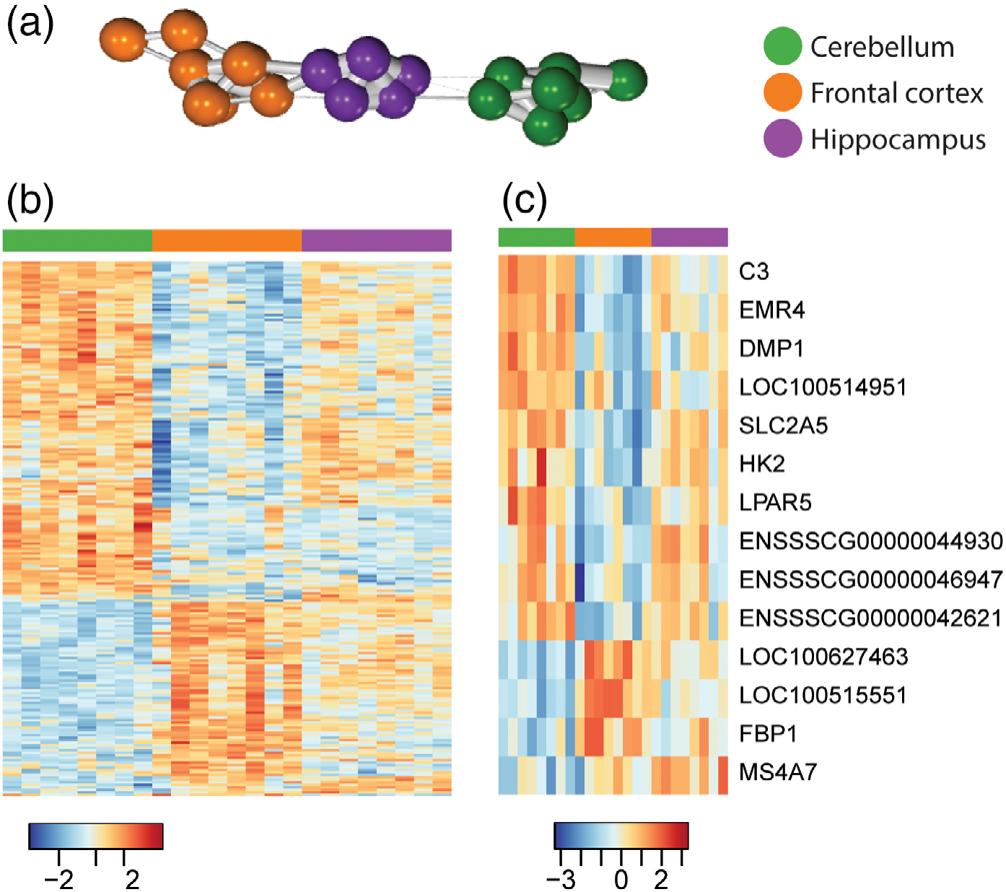
Regional variation of the pig microglial transcriptome. The gene expression for microglia from the frontal cortex (*n* = 8), hippocampus (*n* = 8) and cerebellum (*n* = 8) were compared to each other, and 150 genes show significant regional variation (Table S9). (a) Using the 150 genes showing high regional variation for sample‐sample correlation analysis, microglia from the frontal cortex (orange) and the hippocampus (purple) are clustered closer to each other than to those from the cerebellum (green). (b) Heatmap illustrating the differential expression of the 150 genes across microglia from the three regions. (c) When comparing these 150 genes showing regional variation against the 239 pig microglia‐associated genes, 14 genes are present in both gene lists (Table S10). *Z*‐score was used to represent the FPKM expression as standard deviations away from the mean for each gene.

Studies in mice and humans have reported differences in the transcriptional profiles of microglia from distinct brain regions (Grabert et al., 2016; De Biase et al., 2017; Hammond et al., 2019; Sankowski et al., 2019; Kana et al., 2019). We therefore determined whether the microglia in the pig brain showed regional diversity by comparing microglia from the cerebellum, hippocampus and frontal cortex. This analysis identified 150 genes with significant variation across these regions (adjusted *p* < 0.01; Table S9). Using these genes to carry out sample‐sample correlation analysis suggested that cerebellar microglia were more distinct from microglia in the hippocampus and frontal cortex (Figure 5a). The intermediate profile of hippocampal microglia was similar to previous findings in rodent brain and indicative of a comparable rostro‐caudal gradient in profile (Grabert et al., 2016). GO enrichment analysis on the genes showing regional variation indicated enrichment in biological processes related to negative regulation of leukocyte apoptotic process (GO:2000107) and maintenance of location (GO:0051235) (Figure S2). Of the regionally variant genes, 85 were most highly expressed by cerebellar microglia, 53 genes were most highly expressed by frontal cortex microglia, and 12 were genes most highly expressed by hippocampal microglia (Figure 5b). Among the 150 regionally variant genes, 14 genes overlapped with the 239 pig microglia signature genes (Figure 5c and Table S10) suggesting that differential expression of core identity genes contributes relatively little to regional heterogeneity.

**FIGURE 6:**
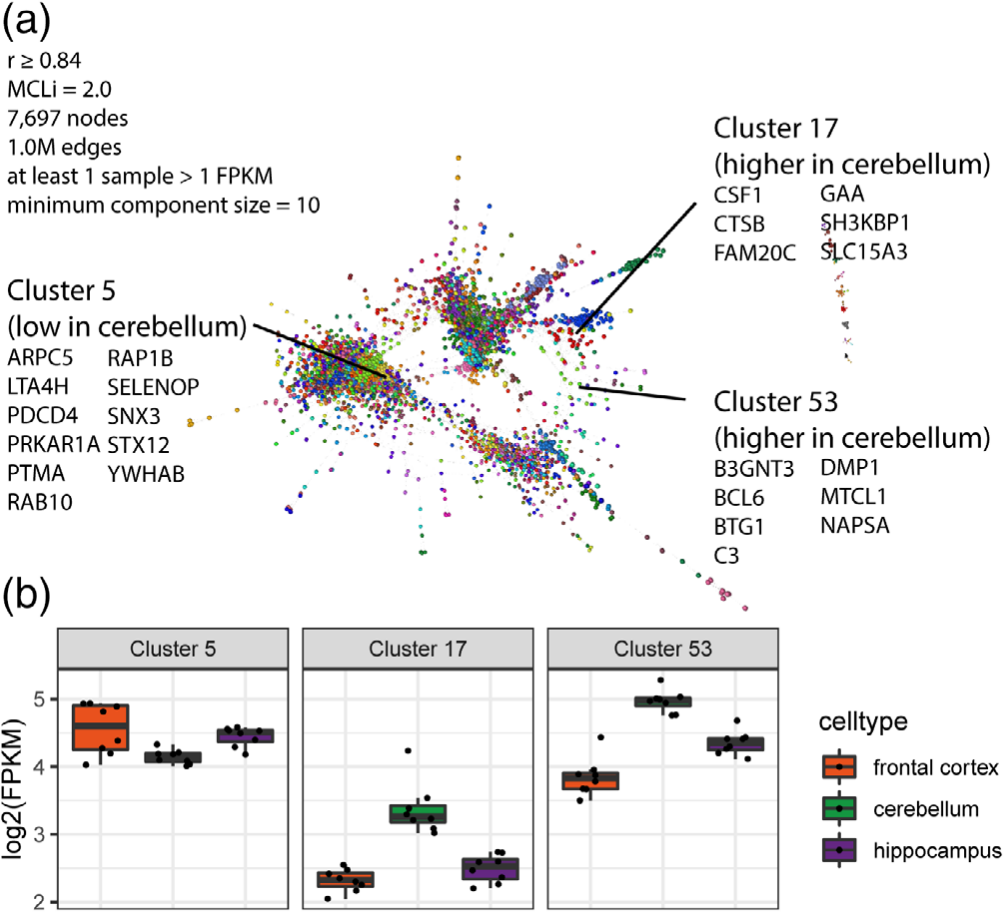
Gene–gene co‐expression network analysis of microglial samples. Co‐expression network analysis was carried out using all pig microglia samples (Table S11). (a) The co‐expression clustering method groups together genes showing similar pattern of expression across samples. Three clusters are found to be enriched in genes showing significant regional variation, Cluster 5, 17, and 53. (b) The average expression levels [log2(FPKM)] for frontal cortex (orange; *n* = 8), cerebellum (green; *n* = 8) and hippocampus (purple; n = 8) for Cluster 5, 17, and 53. Cluster 17 (e.g., *CSF1*, *CTSB*, *SLC15A3*) and 53 (e.g., *C3*, *DMP1*, *NAPSA*) are higher in expression in the cerebellum, while Cluster 5 (e.g., *ARPC5*, *PDCD4*, *RAP1B*) is lower in cerebellum.

**FIGURE 7:**
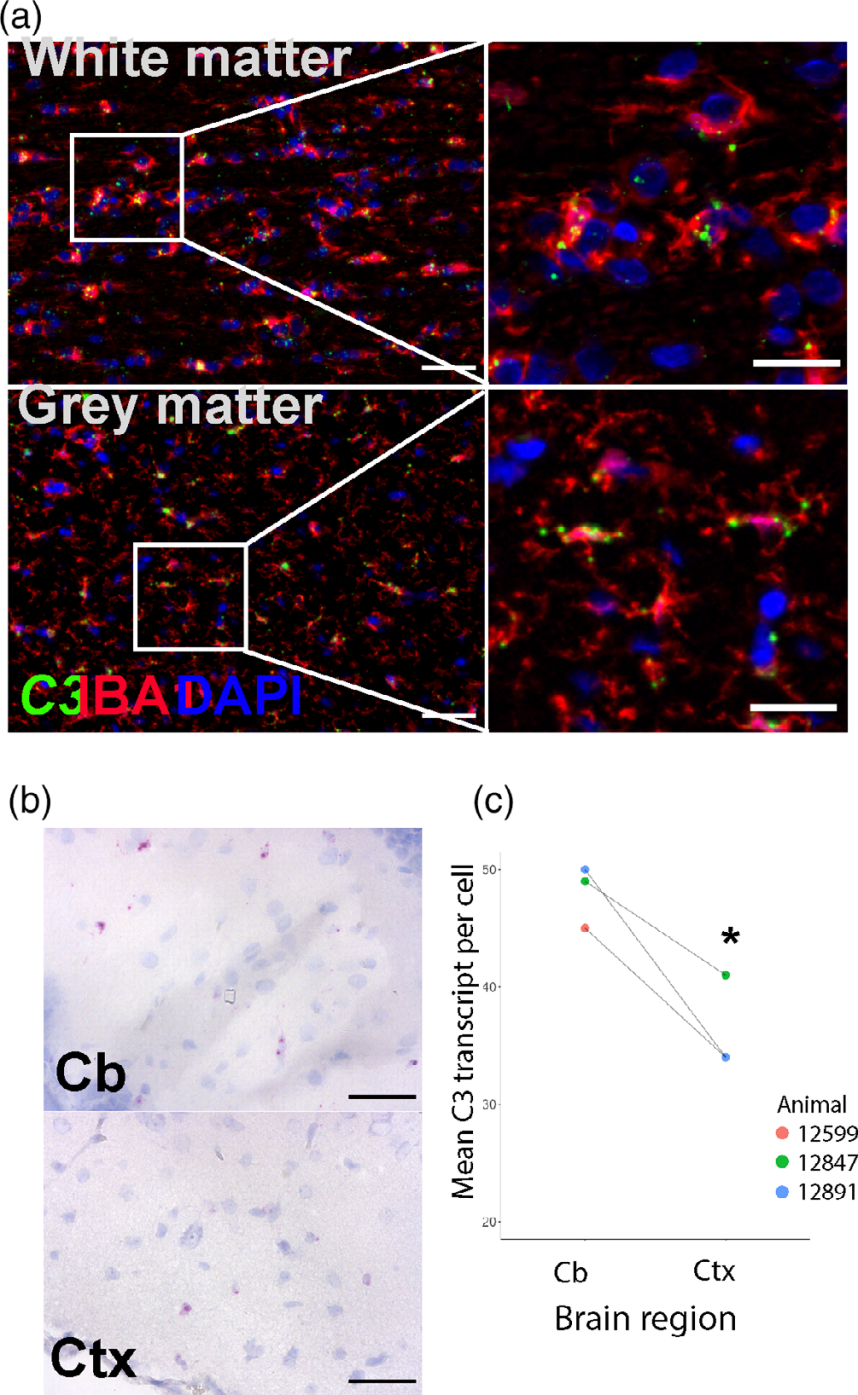
In situ labelling and regional expression of *C3* transcript. *C3* expression was localized to the soma and processes of microglia in both white matter and gray matter regions by coupling fluorescent in‐situ hybridization with immunolabeling of microglial IBA1 (a), scale bars: low mag (left image) = 50 μm, high mag = 25 μm. *C3* (magenta) expression was compared by brightfield microscopy in cerebellar and temporal cortices where low cellularity aids automated image analysis (b), scale bar = 50 μm. *C3* transcript abundance in cerebellar microglia compared to cortical microglia (c), *p* < 0.05; paired t‐test, *n* = 3. Cb = Cerebellum, Ctx = Cortex.

To explore in more depth the biological processes associated with specific regional microglial variation, we examined gene clusters enriched for the 150 genes showing regional‐variation from a co‐expression gene network generated from all genes with at least 1FPKM expression in microglia from the cerebellum, hippocampus and frontal (*r* = 0.84, MCLi = 2.0) (Table S11). The co‐expression network analysis highlighted three co‐expressed gene clusters that were enriched in the 150 regionally variant genes (Figure 6, Table S11): Cluster 5 (lower in cerebellum; e.g., *PDCD4*, *PTMA*, *SNX3*, and *ARPC5*); Cluster 17 (high in cerebellum; e.g., *CTSB*, *CSF1*, and *SLC15A3*); Cluster 53 (high in cerebellum; e.g., *C3*, *BCL6*, and *BTG1*) (Table S11). Cluster 5 genes (relatively higher in frontal cortex and hippocampus) were significantly enriched in GO processes associated with regulation of protein‐containing complex assembly (GO: 0043254, *p* < 10^−5^, Figure S3, e.g., *RHOA*, *CAPZA1*, *H3F3A*, *RAP1B*, *RIOK3*, *TMSB4Y*, *ARPC5*, *P2RY12*, *ARPC5L*, *WASHC2C*), and regulation of protein‐containing complex assembly (GO: 0006397, *p* < 10^−5^, Figure S3, e.g., *HNRNPK*, *MAGOH*, *MFAP1*, *HNRNPM*, *CIR1*, *SF3A3*, *NUDT21*, *SYF2*, *CDC40*, *CPSF3*). Genes in Cluster 17 (relatively higher in cerebellum) were significantly enriched in processes associated with regulation of chemotaxis (GO: 0050920, *p* < 10^−4^, Figure S4, e.g., *CSF1*, *HSPB1*, *LGMN*, *SLAMF8*), whereas Genes in Cluster 53 were not significantly enriched for any GO term (*p* > 10^−4^). We noted that genes associated with complement (e.g., *C3*) and MHC class II (e.g. *CD74*) pathways were among those more highly expressed in cerebellar microglia, similar to our previous findings in mouse microglia assessed by bulk transcriptome and protein‐level analysis (Grabert et al., 2016). Examination of mouse single‐cell RNAseq data from the Dropviz dataset (Saunders et al., 2018) showed higher expression of complement genes and *Cd74* in cerebellar microglia (Figure S5). A recent single nuclear RNAseq profiling of glial cells in the human CNS showed more expression of CD74 and HLA‐DRA in caudal regions compared to BA4 primary motor cortex (Seeker et al., in press) indicating cross‐species conservation of regional microglial heterogeneity and across analytical platforms. We next validated spatial RNAseq profiles of pig microglia by performing in situ hybridization on the highly abundant *C3* gene that was enriched in cerebellar microglia. *C3* colocalised with IBA1+ immunolabeled microglia/macrophages (Figure 7a) and more transcripts per cell were observed in the cerebellum (Figure 7b, c). *C3* labelling was notably more intense within white matter compartments of both cerebellum and frontal cortex. Thus, microglial distribution within white and gray matter across brain regions (e.g. more white matter‐enriched cerebellum) may further contribute to regional heterogeneity when microglia are profiled at bulk population level.

### 3.3 Cross‐species comparison of microglial transcriptional signatures

**FIGURE 8:**
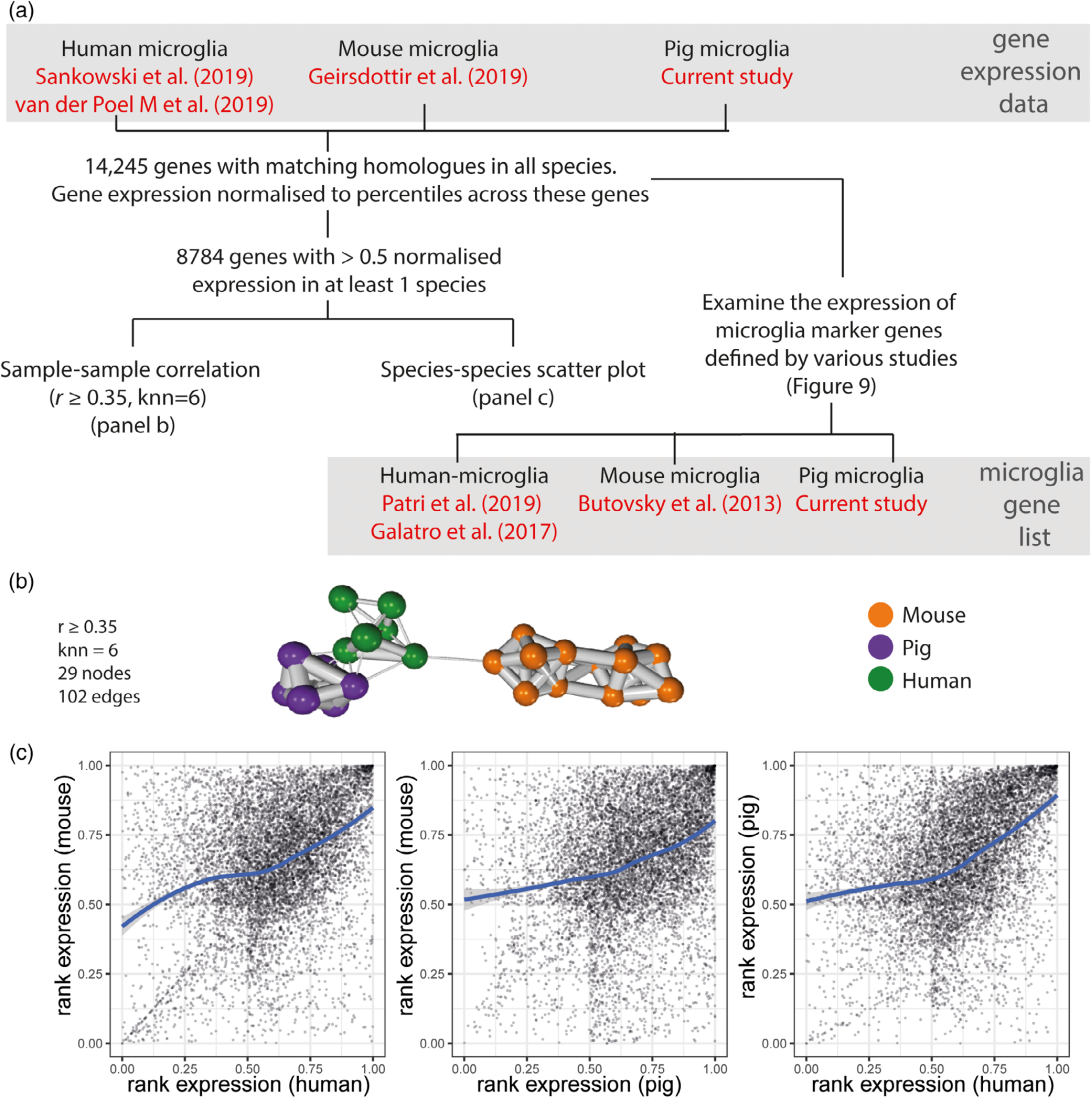
Workflow to compare human, pig, and mouse microglia transcriptomes. (a) Genes with homologues across human, pig and mouse were selected, a total of 14,245 genes. In order to create a combined dataset that can be used to compare across species and data sources, the expression of genes for each sample was normalized to the rank of expression ranged from 0 to 1. Of these, 8784 genes have a normalized rank expression value >0.5 in at least one species (averaged across all samples of the same species) (Geirsdottir et al., 2019; Sankowski et al., 2019; van der Poel et al., 2019). These 8784 gene were used for sample‐sample correlation network analysis (panel b) and scatter plots (panel c). The expression of human (*n* = 6), mouse (*n* = 17) and pig (*n* = 6; two from each brain region) microglia marker genes were also examined across these datasets. Two human microglia gene lists were included, one derived from Patir et al. (2019) and the other from Galatro et al. (2017); the former was based on co‐expression, while the latter was based on differential expression. The mouse microglia gene list was derived from Butovsky et al. (2014), and the pig microglia gene list from the current study. With the exception of the pig microglia data, the gene expression data and the gene lists were from separate studies. (b) Sample‐sample correlation using the normalized dataset, including only genes with >0.5 normalized rank expression in at least 1 species, suggests that overall, human microglia were more similar to pig microglia than those of mouse. (c) Scatter plot smoothing (locally estimated scatterplot smoothing, LOESS) was used for exploring the trend of the relationship between the gene expression for compared species, showing a ratio closer to 1 for genes showing moderate expression (normalized rank expression >0.5) in both species.

The analysis above suggests some degree of species conservation in regional microglial profiles, however we sought to more formally compare the pig microglial transcriptome profile identified here with those in humans and mice. A total of 14,245 genes with a matching human homologue across mice, humans and pigs were identified. To allow cross‐dataset and cross‐species comparison, the RNAseq data were expressed as FPKM and normalized to ranked expression, with 1 being the maximum expression and 0 being the minimum expression (Figure 8). Sample‐sample correlation was then carried out on the normalized combined dataset containing the 8784 genes, including only genes with >0.5 normalized expression in at least one species. This analysis revealed clustering of samples in a species‐related pattern and with noticeably more relatedness (e.g., thicker/shorter/more edges linking sample nodes) within one species than between different species (Figure 8b). The rank order of normalized gene expression for all matching orthologues showed generally well‐conserved expression patterns for genes expressed by both species (i.e., more than 0.5 normalized rank expression) when examining each pairwise comparison of species consistent with sample‐sample correlation analysis (Figure 8c). The Pearson correlation (*r*) between species when using only genes highly expressed by both (>0.5 normalized expression) were 0.36 (human vs. mouse), 0.41 (human vs. pig) and 0.26 (pig vs. mouse).

**FIGURE 9:**
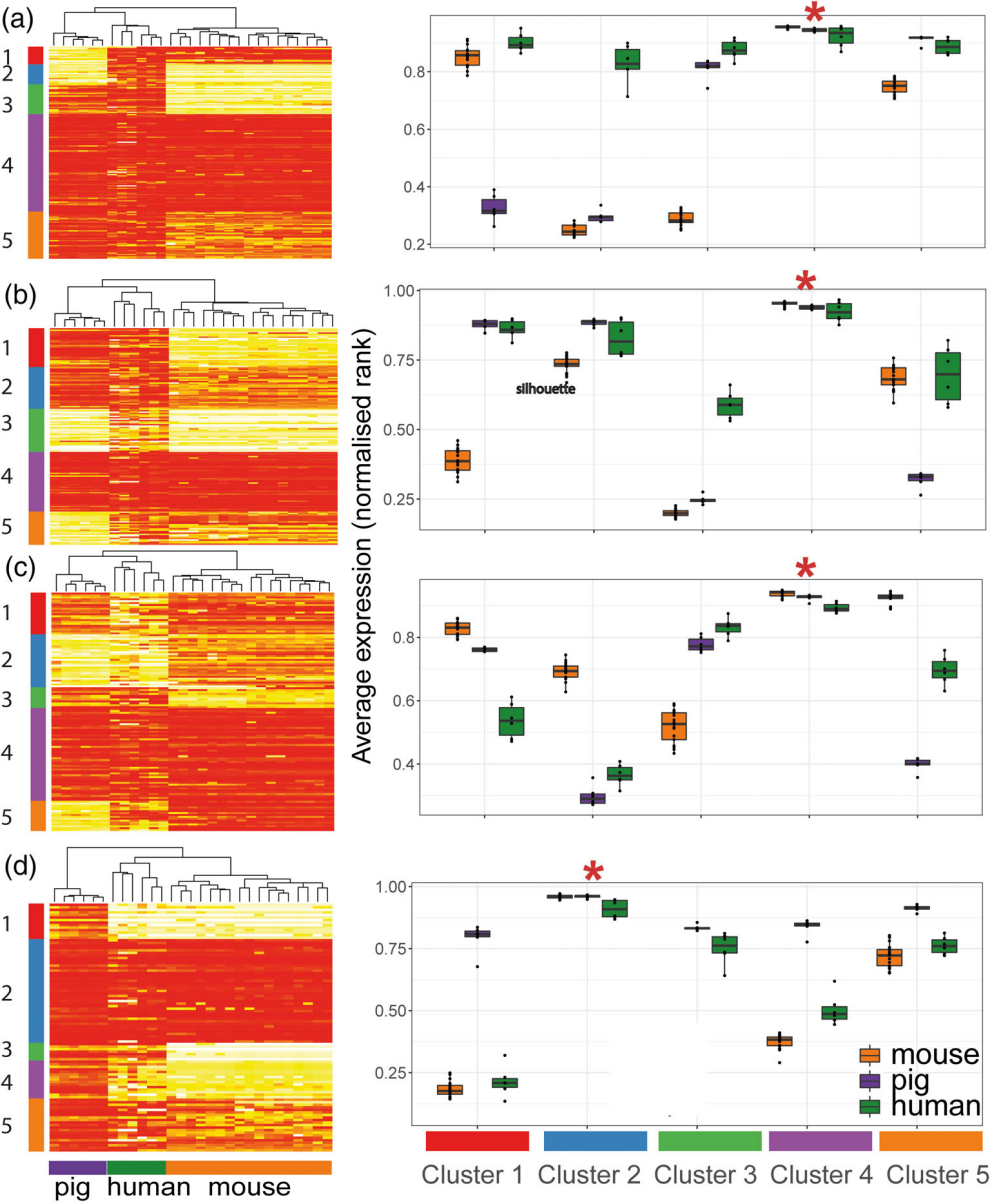
Inter‐species comparison of microglial core signatures. Gene expression data were compared across studies by converting FPKM to normalized rank expression. We then examined the expression of human (*n* = 6), mouse (*n* = 17) and pig (*n* = 6) microglia gene lists in the gene expression data for the three species. By using k‐means clustering, five groups of genes are characterized for each gene list (cluster 1–5), and the average expression for each cluster is shown per species on the right‐hand side of the corresponding heatmaps. (a) Of the human‐microglia genes reported by Patir et al. (2019), 170 genes have matching orthologues in the combined dataset. Cluster 4 is expressed in all three species, whereas Cluster 3 and 5 are less expressed in mouse microglia. Cluster 1 is low in pig and cluster 2 being low in both pig and mouse. (b) Of the 252 human microglia genes reported in Galatro et al. (2017) (additional filters described in methods, 156 genes have matching orthologues in the combined dataset. The observed pattern is similar to analysis done with human microglia markers defined through co‐expression in panel a. (c) Of the 152 mouse microglia genes reported by Butovsky et al. (2014), 126 genes have matching orthologues in the combined dataset. Cluster 4 is expressed in all three species, whereas Cluster 1 is slightly less expressed in human. Cluster 3 is less expressed in mouse microglia. Cluster 5 is low in pig, and cluster 2 is low in both human and pig. (d) Of the 239 pig microglia genes reported in this study, 98 genes have matching orthologues in the combined dataset. Cluster 2 is expressed in all three species, whereas Cluster 3 and 4 are slightly less expressed in mouse. Cluster 5 is expressed in all species, though to a lesser extend in human and mouse. Cluster 1 is low in both human and mouse.

We further explored microglial species relatedness by comparing the expression of microglia signatures gene profiles derived from pigs (this study), human (Galatro et al., 2017; Patir et al., 2019), or mouse (Butovsky et al., 2014) across the cross‐species dataset of 14,245 genes described above (Figures 8 and 9). K‐means clustering was used to group these genes into 5 clusters for each gene list (Figures 8 and 9 and Tables [Link], [Link]). As was expected, for each microglial species‐derived signature gene list, the expression of those signature genes is highest in samples of the same species on the cross‐species dataset (e.g., human microglia show the highest expression for human‐derived microglia genes). This is evident even when the signature gene list and expression data on which the gene list is mapped are derived from different studies. One exception is a small cluster of mouse microglia signature genes (derived from Butovsky et al., 2014) that is not highly expressed by the mouse microglia from Geirsdottir et al. (Cluster 3 in Figure 9c); these may indicate study‐specific genes.

For all species gene signature lists, there is a cluster of genes that is highly and similarly expressed across all species (Figure 9a Cluster 4, Figure 9b Cluster 4, Figure 9c Cluster 4, and Figure 9d Cluster 2). There is a total of 129 unique genes after combining all genes from these clusters (Table S16), 11 of which are present in all four gene lists (*ABI3*, *BLNK*, *CSF1R*, *CX3CR1*, *FCGR1A*, *FGD2*, *GPR34*, *OLFML3*, *P2RY12*, *P2RY13*, *SLC2A5*), which we note include several archetypal microglial signature genes, reaffirming their conserved nature. When examining human microglia genes, some clusters showed lower expression in mouse (Figure 9a Cluster 3 and Figure 9b Cluster 1), while others show lower expression in pigs (Figure 9a Cluster 1, Figure 9b Cluster 5). Several clusters (including Figure 9a Cluster 2, Figure 9b Cluster 3, Figure 9c Cluster 2, and Figure 9d Cluster 1 and 4) were species‐selective with genes expressed highly in samples corresponding to the species from which the gene signature was derived, as expected. Overall, we detected a greater number of clusters and genes showing a more similar level of expression between pig and human microglia than between mouse and human microglia that is reflected in the network clustering and unsupervised hierarchical clustering of samples when using the human‐ and mouse‐derived microglial signature gene lists (Figures 8b and 9).

## 4 DISCUSSION

Here, we conducted the first study to define the pig microglial transcriptome‐wide gene signature and explore regional variation in pig microglial gene expression. Pig microglial identity shares many similarities with both human and mouse microglia, including similar patterns of regional heterogeneity, reinforcing the utility of the pig as a translation‐relevant complementary species for the study of microglia and neuroimmune mechanisms in disease and other challenges to brain homeostasis such as psychosocial stress.

We have reported 239 genes highly enriched in isolated pig microglia relative to macrophages and whole brain tissue from the same brain region. Many of the top 30 pig microglia genes identified in this study have previously been noted as among the most enriched genes when comparing microglia to monocytes/macrophages in multiple mouse genome‐wide gene expression studies, including *P2RY12*, *TMEM119*, *TREM2*, and *CX3CR1* (Butovsky et al., 2014; Chiu et al., 2013; Hickman et al., 2013). *LTC4S* has been validated to be microglia‐enriched in mice (Bennett et al., 2016). SALL1 is not only uniquely expressed by microglia (among adult CNS cell types), but has been found to play a role in the maintenance of microglial identity as its inactivation converts microglia into inflammatory phagocytes (Buttgereit et al., 2016). *TREM2*, *TMEM119*, *CX3CR1*, and *MLXIPL* have also been reported from human studies to be greater in microglia compared to monocytes (Butovsky et al., 2014). *P2RY6* has been demonstrated to be involved in microglial phagocytosis in rat microglia in vitro (Koizumi et al., 2007). *RAB3IL1* and *LTC4S* are found in the full gene list from various human or mice studies examining microglia gene signatures (Bennett et al., 2016; Butovsky et al., 2014; Patir et al., 2019). A recent study showed conservation of a core microglial signature across multiple species (Geirsdottir et al., 2019), although this study did not analyze the pig microglial transcriptome and differing data processing steps precluded a formal comparison with our pig dataset. Thus, microglia have core features that define conserved identity across evolutionary diverse mammalian species, including the pig.

We also examined the variation in gene expression by microglia from different brain regions of the pigs. In additional to acting as immune sentinels, microglia also play also important roles in CNS homeostasis during development and in adult health and disease (Prinz & Priller, 2014). To carry out these multifunctional roles, microglia are required to sense perturbations in their environment to elicit appropriate microglial responses to maintain homeostasis (Baxter et al., 2021; Grabert et al., 2016; Stratoulias et al., 2019). For instance, exposing microglia to signals by healthy neurons and/or astrocytes appears to promote their resting state and antagonize pro‐inflammatory activities (Baxter et al., 2021; Biber et al., 2007). Fractalkine (CX3CR1‐CX3CL1) signaling has been found to regulate microglial activation; neuronal membrane bound CX3CL1 maintains CX3CR1‐expressing microglia in a surveying state, and cleaved soluble CX3CL1 is thought to stimulate migration of inflammatory cells (Szepesi et al., 2018). The mammalian brain is organized into regions with specific biological functions and properties with distinct transcriptional and metabolic profiles (Choi et al., 2018; Hawrylycz et al., 2012). Indeed, microglial regional variation has been described not only in their distribution and morphology (Lawson et al., 1990; Savchenko et al., 1997; Tan et al., 2020), but also in their gene and protein expression in both mice and human (Bottcher et al., 2019; Grabert et al., 2016; Patir et al., 2019; Sjostedt et al., 2020), although the extent of this may depend on the analytical method used. Our study has observed 150 microglial genes showing regional heterogeneity in the pig brain, with 14 such genes also being microglial markers. In this study, we found microglia from the cerebellum to be more distinct from their counterparts from the frontal cortex and hippocampus, which is an observation reported by other studies in rodent and human CNS tissue (Grabert et al., 2016; Seeker et al., in press; Soreq et al., 2017). The finding that only 10% of regionally heterogeneous genes are also part of the core signature gene set is similar to our previous observations in the mouse brain and indicates that regional heterogeneity is primarily superimposed upon core identity (Grabert et al., 2016). Nonetheless, since 14 of these 150 regionally varied genes were also present within our core pig microglia signature gene list, it is important to consider that the expression level of some core genes may vary across the brain when compared to the others and selection of appropriate markers (e.g., for labelling microglia) may require consideration of the regions being analyzed. *C3* was among the core genes that did show regional heterogeneity and more broadly this was representative of elevated complement gene expression in cerebellar microglia compared to other brain regions, a pattern also observed in mouse by bulk (Grabert et al., 2016) and single cell RNA sequencing (Figure S5 (Saunders et al., 2018)) and for other pathways such as MHC class II (e.g., *CD74*). A recent human study demonstrating greater microglial activation status (e.g., *CD74* expression elevated) in human spinal cord microglia adds to the evidence suggesting increasing microglial immune alertness progressing along the rostrocaudal neuroaxis in multiple species (Seeker et al., in press).

When comparing the expression of microglial gene signatures derived from human, mouse, and the present pig datasets, there is a group of microglial genes that are highly expressed by microglia of all three species. Indeed, this formed the largest cluster irrespective of the source species of the signature, highlighting that overall there are more similarities than difference among the species. However, based on unsupervised clustering from the cross‐species analysis there was some indication that pig and human microglia have similarly high expression of a greater subset of signature genes as compared to mouse and human. We noted this included genes relating to the complement system, including complement components *C2* and *C3*. Geirsdottir et al. (2019) have noted complement genes are expressed at a lower level in rodent microglia than human microglia. Galatro et al. (2017) have highlighted several immune genes such as *TLR*, *Fc*, and *SIGLEC* receptors, to be abundantly expressed in human microglia but not in mouse microglia. When comparing pig, human and mouse microglia, we found groups of genes showing higher level of expression in human and pig microglia compared to the mouse, including *TLR1*, *TLR3*, *TLR6*, *FCGR3B*, *SIGLEC10*, and *SIGLEC11*. TLR3 expression has been positively correlated with plaques in Alzheimer disease as well as colocalising with the phagocytic marker CD68 (Walker et al., 2018). Although more human microglia markers are expressed by pig microglia than mouse microglia, there is a subset of genes that is more similar between mouse and human microglia, and each species exhibit species‐specific markers. It is possible that unavoidable differences in methods for microglial isolation in different species/studies may influence the extent of species microglial relatedness, however our pig isolation protocol is similar to commonly used rodent protocols and we saw similar cross‐species patterns when the human microglial signature was derived from different studies using distinct methods (e.g. biopsy or post‐mortem samples). By comparison with an immediate early gene list (Vacca et al., 2018), we found no overlap with our pig microglial core gene signature, indicating that aberrant activation, which can occur during tissue disaggregation, was also unlikely a contributor to measures of species relatedness. Noting the differences as well as the similarities in gene expression pattern across microglia from different species highlights the importance of characterizing pig‐specific microglial signatures for facilitating a better understanding of pig neuroimmunology and pathology, and utility of the pig in translational biomedical and agricultural research.

In conclusion, we have defined the pig microglia transcriptome signature that distinguishes microglia from other CNS cell types and non‐CNS macrophages, proposing gene sets that can be used for differentiating the different myeloid cell types in the pig. We have demonstrated regional variation in pig microglial gene expression, with those derived from the cerebellum being more distinct from those from the frontal cortex and hippocampus. Our results indicate that pig, human, and rodent microglia share common transcriptome‐wide and signature profiles overall, although our data suggest the expression of a portion of genes relating to complement and antigen presenting pathways may be more similar in pig and human microglia.

## AUTHOR CONTRIBUTIONS

Conceptualization: SMB, GT, ABL, BWM; Methodology: all authors; Investigation: all authors; Analysis: all authors; Supervision: NAM, JP, GT, ABL, BWM; Project administration: NAM, JP, GT, ABL, BWM; Writing – original draft: all authors; Writing – review and editing: all authors; Funding acquisition: SMB, GT, ABL, BWM.

## Supporting information


**Figure S1** FACS gating for cell isolation
Flow cytometry of single cell suspensions derived from brain or lung. Each plot shows the fluorescence intensities of events corresponding to the fluorophore on the axis. Gates used for selection are displayed as black outline boxes. Selection criteria for microglia (CD11b^+^ CD45^lo^) and macrophage (F4/80^+^) are as described in the Flow Cytometry section in Methods. (a) Unstained brain cell suspension. Minimal recorded events within the gate can be attributed to background fluorescence; (b) Stained brain cell suspension with microglia identified in the gated area in purple; (c) Unstained alveolar lavage cell suspension. Minimal recorded events within the gated area attribuTable to background; (d) Stained alveolar lavage cell suspension with macrophages identified in the gate in red.

**Figure S2** Pathway enrichment analysis for genes showing regional variation
Metascape was used for carrying out pathway enrichment analysis on genes showing significant regional variation (TableS9) using Gene Ontology (GO) Biological Processes database. Negative regulation of leukocyte apoptotic process (GO:2000107) and maintenance of location (GO:0051235) were the most significant pathways.

**Figure S3** Pathway enrichment analysis for genes showing regional variation
Metascape was used for carrying out pathway enrichment analysis on genes in Cluster 5 in the gene–gene network analysis (TableS11) using Gene Ontology (GO) Biological Processes database. This cluster was enriched in genes showing lower expression in cerebellum. Regulation of protein‐containing complex assembly (GO: 0043254) and mRNA processing (GO:0006397) were the most significant pathways.

**Figure S4** Pathway enrichment analysis for genes showing regional variation
Metascape was used for carrying out pathway enrichment analysis on genes in Cluster 17 in the gene–gene network analysis (TableS11) using Gene Ontology (GO) Biological Processes database. This cluster was enriched in genes showing higher expression in cerebellum. Regulation of chemotaxis (GO:0050920) was the most significant pathway.

**Figure S5** Expression of CD74 and C3 in DropViz
The expression of *C3* and *CD74* in mouse microglia were obtained from DropViz (http://dropviz.org/) (Saunders et al., 2018). *C3* was not detected in cerebellum and hippocampus from mice microglia, and the level of *C3* across mouse microglia were low across all regions (less than 1 transcript per 100,000 in cluster), which could reflect the species variation. A higher level of *CD74* is seen in cerebellum for both pig and mouse, albeit the difference appears larger for the mouse microglia data from DropViz.
The reported confidence intervals in the upper panel reflect statistical sampling noise (reflecting total number of UMIs ascertained by cluster) rather than cell–cell heterogeneity.

**Table S1** highly expressed in microglia vs macrop

**Table S2** highly expressed in microglia vs tissue

**Table S3** DEG removed based on brainrnaseq

**Table S4** pig microglia enriched genes stringent

**Table S5** top30 genes microglia

**Table S6** highly expressed in macrophage and other immune

**Table S7** highly expressed in macrophage

**Table S8** top30 genes macrophage

**Table S9** regional variation

**Table S10** regional pig microglia

**Table S11** full geneset network microglia mcl

**Table S12** kmeans cluster patir

**Table S13** kmeans cluster galatro

**Table S14** kmeans cluster butovsky

**Table S15** kmeans cluster pig

**Table S16** genes in high all species clusters present once

## Data Availability

The RNA‐seq data is available via Gene Expression Omnibus (GEO https://www.ncbi.nlm.nih.gov/geo/, accession number: GSE172284).
