# Supplementary figures and images for: Defining the pig microglial transcriptome reveals its core signature, regional heterogeneity, and similarity with human and rodent microglia
Source: Glia. 2022 Sep 19;71(2):334–49. doi: 10.1002/glia.24274 (PMC10087207; doi:10.1002/glia.24274)

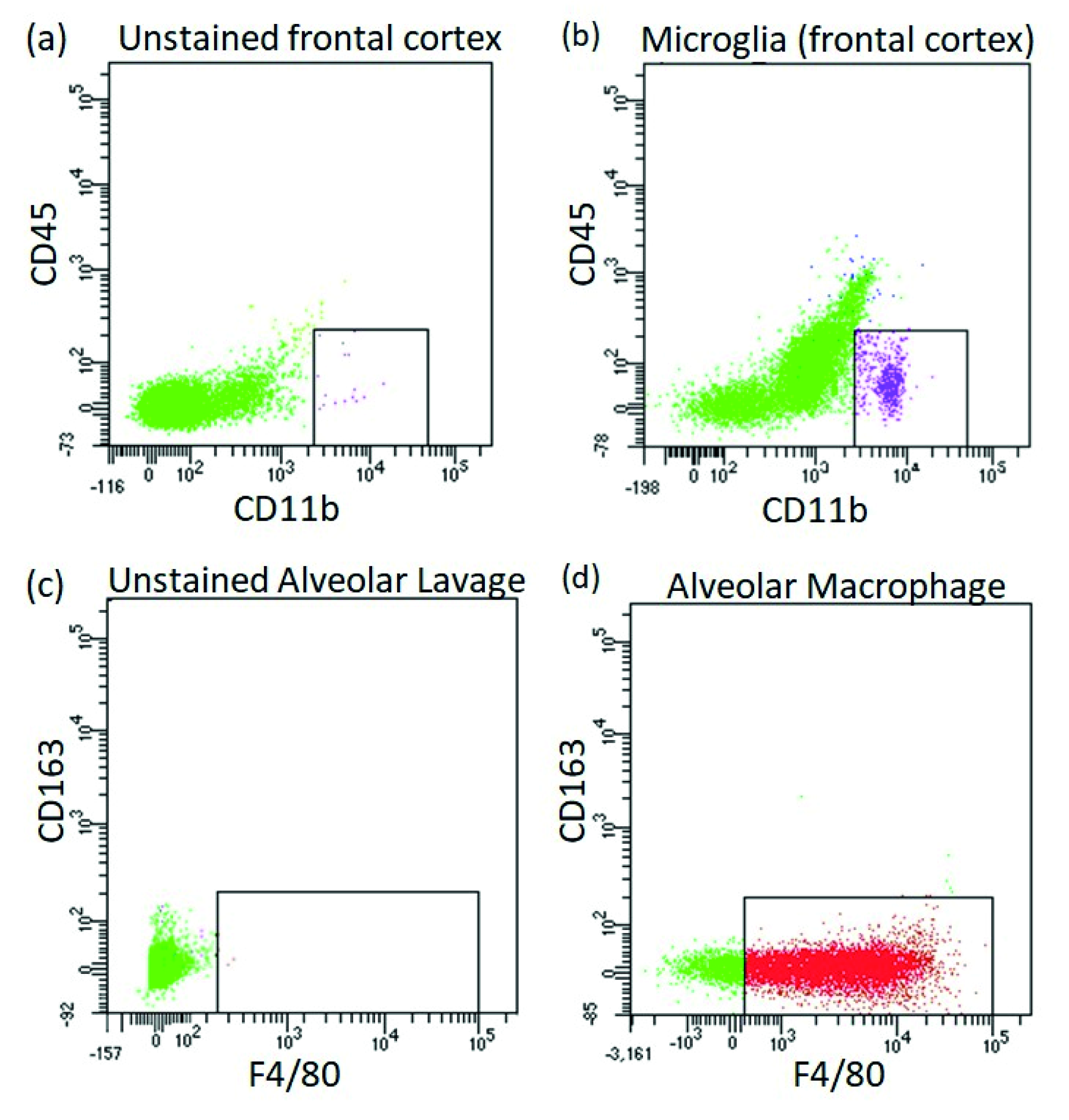

Supplement: Supplementary file 1 — Figure S1 FACS gating for cell isolation Flow cytometry of single cell suspensions derived from brain or lung. Each plot shows the fluorescence intensities of events corresponding to the fluorophore on the axis. Gates used for selection are displayed as black outline boxes. Selection criteria for microglia (CD11b+ CD45lo) and macrophage (F4/80+) are as described in the Flow Cytometry section in Methods. (a) Unstained brain cell suspension. Minimal recorded events within the gate can be attributed to background fluorescence; (b) Stained brain cell suspension with microglia identified in the gated area in purple; (c) Unstained alveolar lavage cell suspension. Minimal recorded events within the gated area attribuTable to background; (d) Stained alveolar lavage cell suspension with macrophages identified in the gate in red. [file GLIA-71-334-s012.tif]

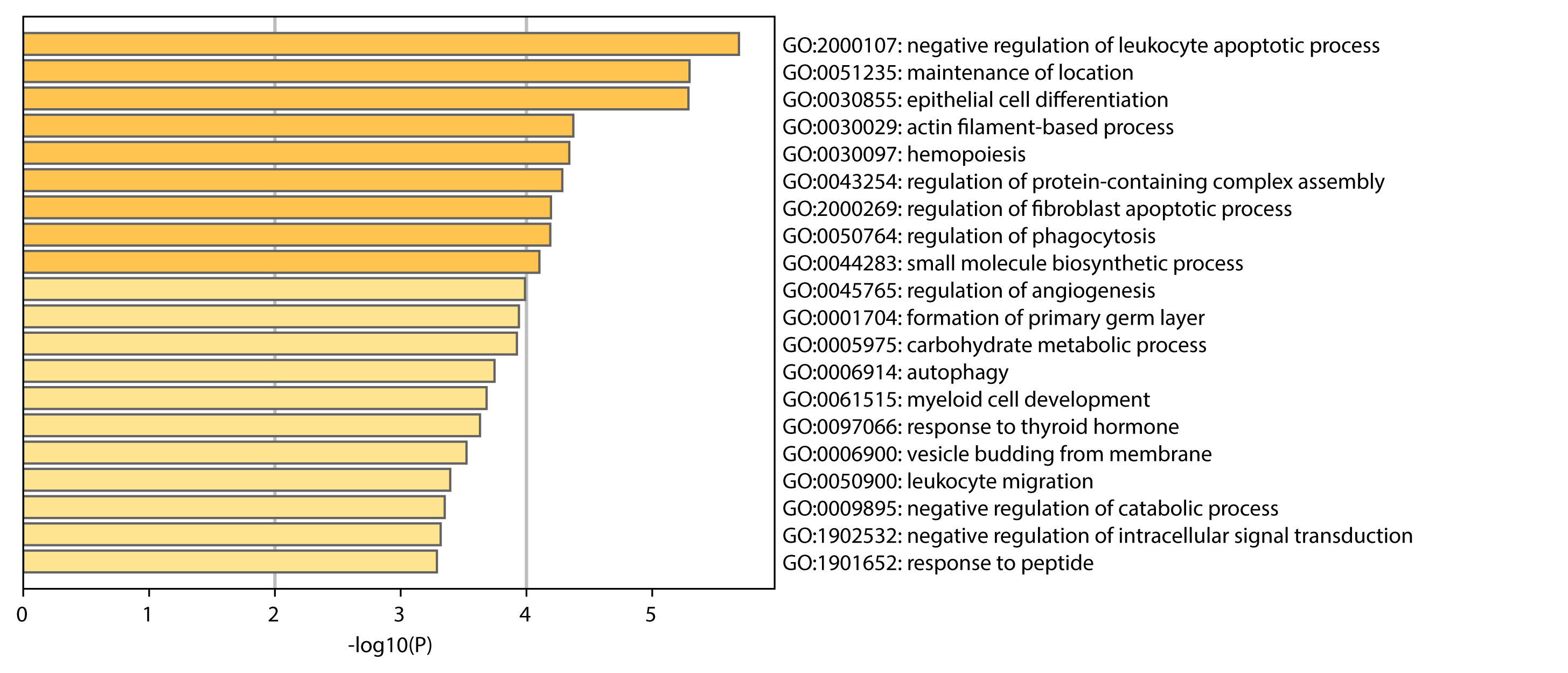

Supplement: Supplementary file 2 — Figure S2 Pathway enrichment analysis for genes showing regional variation Metascape was used for carrying out pathway enrichment analysis on genes showing significant regional variation (TableS9) using Gene Ontology (GO) Biological Processes database. Negative regulation of leukocyte apoptotic process (GO:2000107) and maintenance of location (GO:0051235) were the most significant pathways. [file GLIA-71-334-s007.tif]

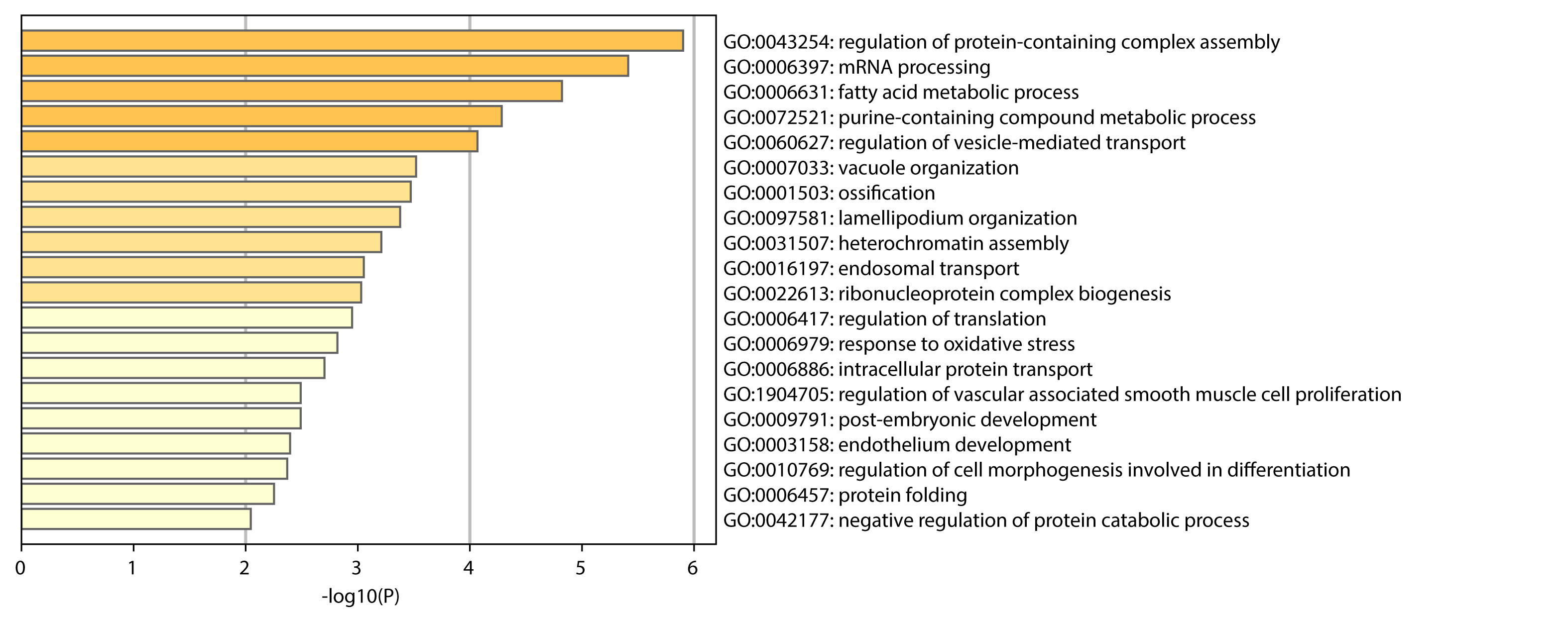

Supplement: Supplementary file 3 — Figure S3 Pathway enrichment analysis for genes showing regional variation Metascape was used for carrying out pathway enrichment analysis on genes in Cluster 5 in the gene–gene network analysis (TableS11) using Gene Ontology (GO) Biological Processes database. This cluster was enriched in genes showing lower expression in cerebellum. Regulation of protein‐containing complex assembly (GO: 0043254) and mRNA processing (GO:0006397) were the most significant pathways. [file GLIA-71-334-s005.tif]

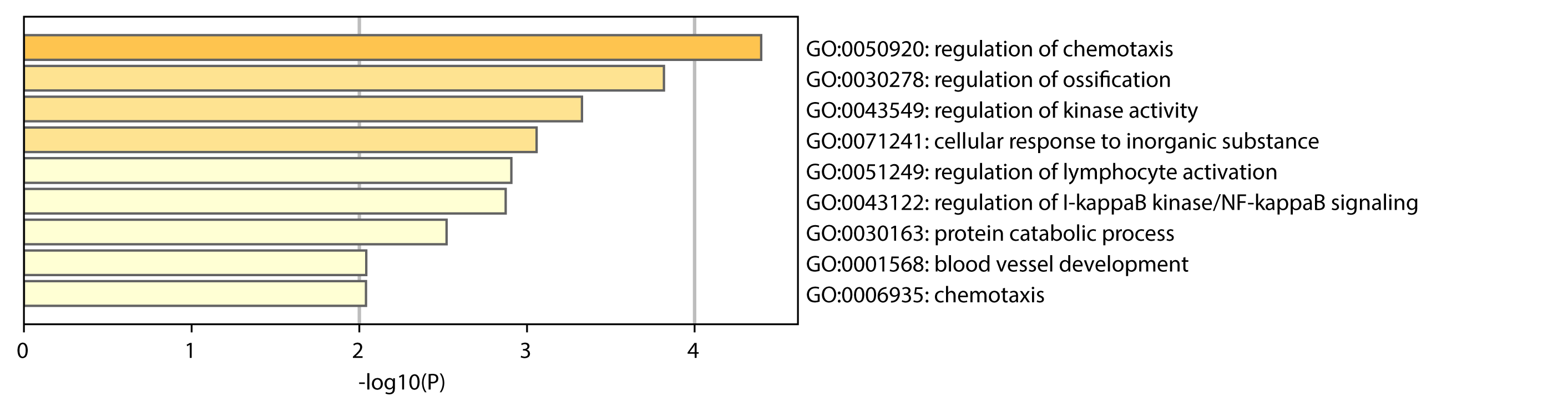

Supplement: Supplementary file 4 — Figure S4 Pathway enrichment analysis for genes showing regional variation Metascape was used for carrying out pathway enrichment analysis on genes in Cluster 17 in the gene–gene network analysis (TableS11) using Gene Ontology (GO) Biological Processes database. This cluster was enriched in genes showing higher expression in cerebellum. Regulation of chemotaxis (GO:0050920) was the most significant pathway. [file GLIA-71-334-s016.tif]

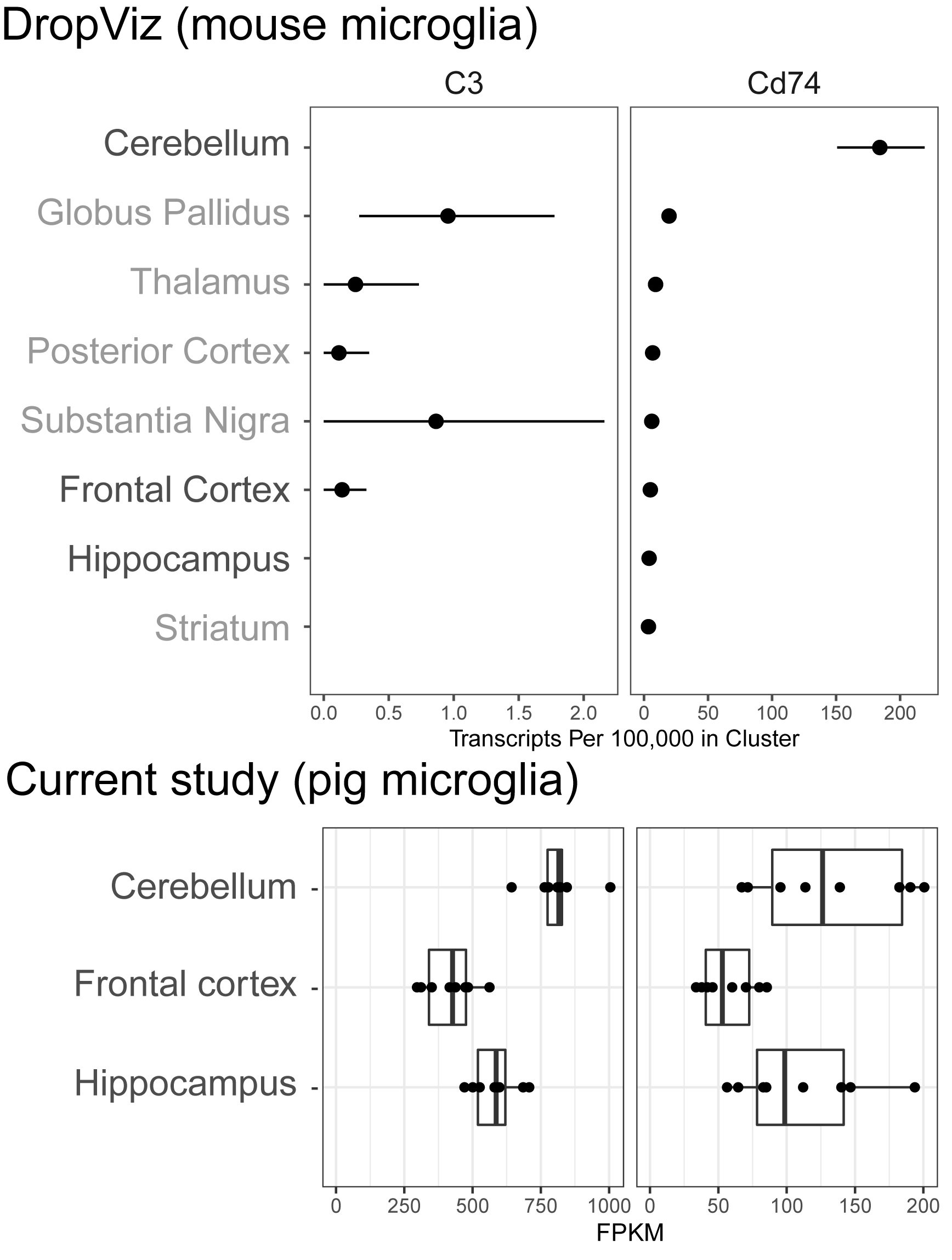

Supplement: Supplementary file 5 — Figure S5 Expression of CD74 and C3 in DropViz The expression of C3 and CD74 in mouse microglia were obtained from DropViz (http://dropviz.org/) (Saunders et al., 2018). C3 was not detected in cerebellum and hippocampus from mice microglia, and the level of C3 across mouse microglia were low across all regions (less than 1 transcript per 100,000 in cluster), which could reflect the species variation. A higher level of CD74 is seen in cerebellum for both pig and mouse, albeit the difference appears larger for the mouse microglia data from DropViz. The reported confidence intervals in the upper panel reflect statistical sampling noise (reflecting total number of UMIs ascertained by cluster) rather than cell–cell heterogeneity. [file GLIA-71-334-s002.tif]
